# Association Between Electronic Device Usage, Physical Activity, and Sleep Quality Related to Cervicogenic Headache Among College Students in Saudi Arabia

**DOI:** 10.3390/healthcare14121695

**Published:** 2026-06-13

**Authors:** Shahul Hameed Pakkir Mohamed, Abdulaziz A. Albalwi, Mohamed Taher Mahmoud Eldesoky, Hamad S. Al Amer, Ahmad A. Alharbi, Jana Alhmeed, Emtenan Alhakami, Shahad Battal Alanazi, Maha Alrashedi, Ghala Dakhilallah

**Affiliations:** Department of Health Rehabilitation Sciences, Faculty of Applied Medical Sciences, University of Tabuk, Tabuk 71491, Saudi Arabia; aa-albalwi@ut.edu.sa (A.A.A.); meldesoky@ut.edu.sa (M.T.M.E.); halamer@ut.edu.sa (H.S.A.A.); aaalharbi@ut.edu.sa (A.A.A.); 431004341@st.ut.edu.sa (J.A.); 441002282@stu.ut.edu.sa (E.A.); 441000781@st.ut.edu.sa (S.B.A.); 431001939@st.ut.edu.sa (M.A.); 441000824@st.ut.edu.sa (G.D.)

**Keywords:** headache disorders, neck pain, posture, exercise, screen time, sleep quality, students

## Abstract

**Background and Objectives:** Cervicogenic headaches (CGH) are increasingly common among college students and may negatively affect academic performance and sleep quality. This study aimed to identify the self-reported prevalence of cervicogenic-type head and neck pain in a convenience sample of Saudi college students and to examine its associations with electronic device use, physical activity, and sleep quality among college students in Saudi Arabia. **Materials and Methods:** A cross-sectional study was conducted among 313 college students from various Saudi university colleges using an online self-administered questionnaire. The questionnaire gathered information on sociodemographic characteristics, electronic device usage, neck pain awareness, physical activity levels, and sleep quality. Descriptive statistics were used to summarize the data, and chi-square tests were used to explore associations between potential predictors and the prevalence of self-reported cervicogenic-type head and neck pain consistent with possible CGH. **Results:** Most participants were female (84.3%) and aged 18–25 years (95.2%). Cervicogenic-type head and neck pain were reported by 65.2% (n = 204/313), while 56.5% experienced moderate to severe stress. A significant association was found with perceived stress (*p* = 0.002). Prolonged electronic device use (>4 h/day: 77.9%; *p* < 0.01), lower physical activity (*p* = 0.056), medication use (*p* < 0.01), headache exacerbation with inactivity (*p* = 0.006), and poor sleep quality (95.1% with PSQI > 10; *p* = 0.044) were significantly associated. **Conclusions:** These findings highlight associations between excessive electronic device use, low physical activity, and poor sleep quality with self-reported cervicogenic-type head and neck pain among Saudi college students. Future longitudinal studies and randomized controlled trials are needed to determine whether targeting these factors reduces the prevalence of CGH.

## 1. Introduction

Cervicogenic headache (CGH) is a secondary headache that originates in the posterior neck and radiates to the frontal region of the head, and the ipsilateral eye, and the duration of this headache can range from several hours to days [1]. CGH shares overlapping clinical features with migraine and tension-type headache, complicating differential diagnosis [2]. Emerging evidence indicates that migraine, tension-type headache, and CGH share common cervical musculoskeletal impairments and mechanisms related to peripheral and central sensitization, including altered trigeminocervical nociception and sensitized peripheral afferents [3,4]. Cervical musculoskeletal dysfunction—such as reduced range of motion, segmental hypomobility, and increased pressure pain thresholds—has been documented across headache types, suggesting that assessment of posture, neck pain, and device-related postural strain is warranted regardless of headache classification [3]. Various factors, such as fatigue, disrupted sleep, cervical disc disorders, neck trauma, poor posture, muscle strain, and smoking, have been associated with CGH [5]. A recent bibliometric review found that cervicogenic headache is a common condition, with prevalence estimates ranging from 0.4% to 42% [6]. In Saudi Arabia, the incidence of headaches was reported at 44.2%, exceeding many other common health issues [7].

Smartphone use has grown rapidly over the past decade, with global penetration estimated at approximately 41.5% and nearly universal adoption among young adults in several regions, a trend that has substantially increased exposure to prolonged screen-based postures associated with cervical strain [8,9]. Adults, including college students, spend significant time using smartphones, tablets, and computers, often with prolonged static postures that place mechanical load on the cervical spine, a key contributor to cervicogenic-type headaches [10]. Prolonged smartphone use promotes forward head posture (FHP), in which the head translates anteriorly relative to the cervical spine. This posture leads to shortening and increased tone of the suboccipital muscles while simultaneously placing the cervical extensor muscles under sustained eccentric load. Separately, acute neck flexion during downward gaze at a screen elongates the suboccipital structures and increases compressive loads on the posterior cervical joints, both mechanisms contributing to neck pain and cervicogenic-type headache [11,12]. It is crucial to investigate the underlying causes and implement early interventions to address improper posture [13]. Saudi medical students may be more vulnerable to device-related headaches due to the growing use of technology in medical education [14]. Excessive mobile phone use has been associated with increased headache frequency, likely mediated through cervical postural strain, eye strain, and sleep disruption [15]. These mechanisms warrant early clinical investigation and preventive intervention. Nevertheless, there is a dearth of thorough studies examining various aspects of headaches, including patterns, causes, severity, and knowledge gaps specific to Saudi college students. This issue is troubling, as the particular stresses and strains of medical school may make students more susceptible to headaches triggered or exacerbated by extended use of electronic devices for learning [16].

Numerous behavioral and lifestyle approaches, such as stress reduction, dietary modifications, improved sleep hygiene, and increased physical activity, have been investigated for the prevention and treatment of migraines [17]. Studies on physical activity levels in the general population of Saudi Arabia indicate that a significant proportion does not meet the recommended physical activity guidelines [18]. Previous Saudi-based research has established associations between screen time and headaches in university students; Abou Hashish et al. (2022) reported significant screen-related headaches among Saudi health sciences students during the COVID-19 pandemic [19]. While the majority of exercise intervention studies have focused on migraine, the underlying mechanisms, including endorphin release, stress reduction, and improved sleep quality, are physiologically relevant to cervicogenic-type headaches as well, given the shared musculoskeletal and neurological pathways [20]. Yoga and aerobic exercise are also used to reduce migraine attacks and alleviate their symptoms in a new clinical practice guideline [20]. Additionally, this guideline offered particular recommendations for exercise prescriptions based on clinical study criteria.

Poor sleep quality is a recognized risk factor and perpetuating factor for CGH, as sleep deprivation lowers pain thresholds and increases muscle tension in the cervical region [21,22]. Among college students, disrupted sleep hygiene adversely affects cognitive function, academic performance, and pain sensitivity [23]. Given that neck pain secondary to poor posture and device use may itself impair sleep, investigating the bidirectional relationship between sleep quality and CGH in this population is warranted. Identifying associations between electronic device use, physical activity, sleep quality, and self-reported head and neck pain may help inform educational initiatives and clinical screening approaches targeting modifiable risk behaviors in college students.

Despite growing evidence linking electronic device use to neck pain and headache, several important gaps remain. First, most existing studies have examined headache broadly rather than focusing specifically on cervicogenic-type presentations involving co-occurring head and neck pain. Second, the joint contribution of device use, physical activity, and sleep quality to CGH burden has rarely been examined within a single study. Third, and critically, no prior study has investigated these associations specifically among college students in Saudi Arabia, a population with high device dependency, limited ergonomic awareness, and documented physical inactivity [21,22]. The present study was designed to address these gaps by examining the prevalence of self-reported cervicogenic-type head and neck pain and its associations with the above lifestyle and behavioral factors in a sample of Saudi college students. This study aimed to estimate the self-reported prevalence of cervicogenic-type head and neck pain in a convenience sample of Saudi college students and to examine its associations with electronic device use, medication use, physical activity, and sleep quality.

## 2. Methods

### 2.1. Study Design and Collection of Data

This cross-sectional study was carried out during April 2026 using a self-structured questionnaire in Arabic, translated via forward translation. The questionnaire was distributed electronically via Google Forms to college students enrolled at five government universities in different regions of Saudi Arabia (Southern, Central, Eastern, Western, and Northern). Distribution used university student group channels (WhatsApp and university e-mail lists) with the assistance of academic staff liaisons at each institution. Participation was voluntary, unpaid; no incentives of any kind were provided. Consent was obtained prior to data collection. As the questionnaire was distributed electronically through student communication channels with an unknown total reach, an overall response rate could not be determined, limiting assessment of non-response bias.

### 2.2. Inclusion and Exclusion Criteria

The study was open to university students who were willing to participate, regardless of their college year. Exclusions were made for ineligible individuals, including those who refused to participate, were under the age of 18, had a history of neurological disorders, had previously experienced a cervical spine injury or surgery, or had been diagnosed with chronic migraines, among other criteria.

### 2.3. Self-Structured CGH Questionnaire Tool

Participants completed a 37-item questionnaire covering electronic device usage, headache patterns, physical activity, and sleep quality. Participants were identified as having possible CGH based on self-reported concurrent head and neck pain. This classification was not based on ICHD-3 diagnostic criteria, which require clinical assessment, including provocation by neck movement or palpation, restricted cervical range of motion, and unilateral pain without side-shifting. This represents a key limitation of the study design, and future work should incorporate clinical examination or validated diagnostic criteria to confirm CGH. The CGH questionnaire tool was self-structured, with items drawn from two prior Saudi-based studies [21,22]. These source studies used item pools informed by existing headache and screen-time literature, but did not independently validate their instruments using factor analysis or item-response theory. The final questionnaire used in the present study was not subjected to formal psychometric testing prior to administration. The Pittsburgh Sleep Quality Index (PSQI), which formed the final section of the questionnaire, is a validated, internationally recognized instrument with established reliability (Cronbach’s alpha = 0.83) and has been validated in an Arabic version [24]. The absence of psychometric validation for the non-PSQI components is acknowledged as a limitation, and future studies should develop and validate a dedicated Arabic-language CGH screening questionnaire for use in Saudi populations.

### 2.4. The Pittsburgh Sleep Quality Index

The Pittsburgh Sleep Quality Index (PSQI) assesses seven aspects of sleep patterns, including bedtimes, wake-up times, total sleep duration, sleep efficiency, sleep onset latency, and daytime dysfunction over the preceding month. Scores ≥ 5 or ≥8 indicate poor or extremely poor sleep quality on the global scale (0–21) [25]. The PSQI is a globally validated instrument (Cronbach’s alpha = 0.83) with an established Arabic version [24].

### 2.5. Sample Size Calculation

The required sample size was estimated using the formula for a single proportion: n = Z^2^·p·(1 − p)/e^2^, where Z = 1.96 (95% confidence level), p = 0.50 (conservative prevalence estimate), and e = 0.05 (5% margin of error), yielding a minimum of 384 participants. After accounting for potential non-responses and exclusions, a target of approximately 325 participants was set, with 313 meeting the eligibility criteria.

### 2.6. Statistical Analysis

Descriptive statistics summarized data by number and percentage. Chi-square tests of independence were used to examine associations between cervicogenic headache status and categorical variables, including perceived stress, electronic device use patterns, physical activity, medication use, and sleep quality. This approach assesses whether the distribution of responses differs across headache severity categories, consistent with the study’s aim of identifying associations rather than causal relationships. A *p*-value < 0.05 was considered significant. Data analysis used SPSS statistic software (Version 25.0; IBM Corp., Armonk, NY, USA).

## 3. Results

**Figure 1:**
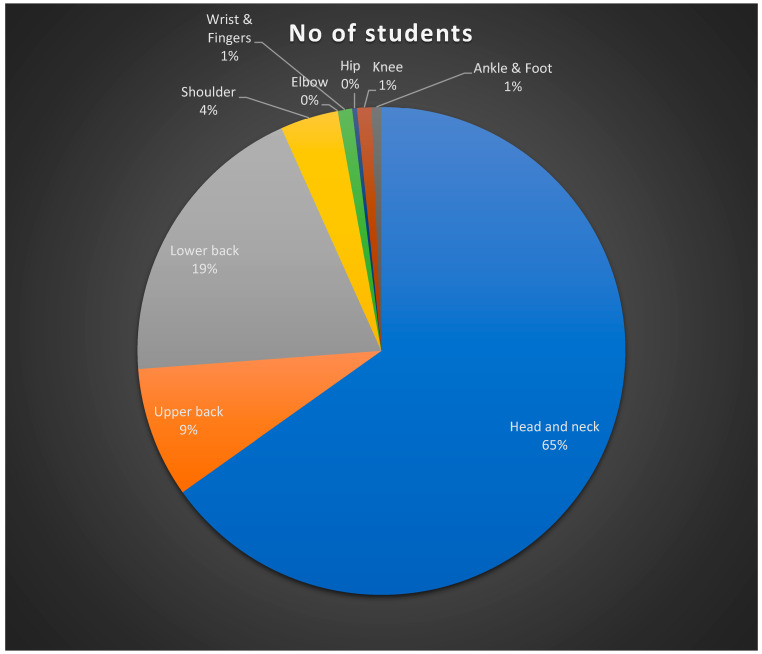
Painful body regions among the college students.

The current study included 325 participants, of whom 12 were excluded based on the established exclusion criteria. The study included 313 eligible participants; most were female (n = 264, 84.3%). Most were aged 18–25 years (n = 298, 95.2%), predominantly single (n = 295, 94.2%), and in their third to fifth year (n = 187, 59.7%). A majority reported head and neck pain (n = 204, 65.2%), followed by lower back (n = 61, 19.5%) and other areas (n = 48, 15.3%) (Table 1) and (Figure 1). More than half reported moderate-to-severe perceived stress (n = 197, 56.5%).

**Figure 2:**
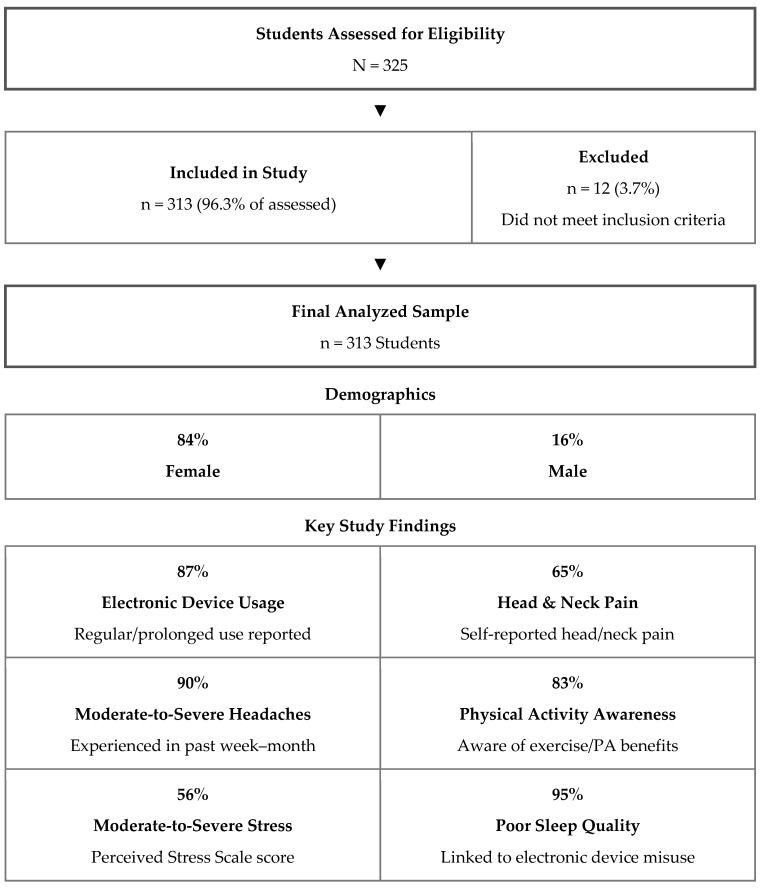
Flowchart of participant recruitment and characteristics for the cross-sectional study.

For the purpose of examining associations with self-reported cervicogenic-type head and neck pain, only participants who reported head and neck pain (n = 204, 65.2%) were included in subsequent analyses (Table 2, Table 3, Table 4 and Table 5), and Figure 2 presents a flowchart of participant recruitment and characteristics for the cross-sectional study. Participants reporting pain exclusively in other regions were not classified as having possible CGH and were excluded from these analyses. Among those with head and neck pain, the most used devices were smartphones and tablets (88.2%), and 159 participants (77.9%) reported using devices for more than 4 h daily.

A significant number (90.7%) reported moderate-to-severe headaches over the past week to a month. Approximately half (50%) used headache medications, while 55.4% could tolerate a headache without medication for less than 30 min. None reported headaches after exercising. Light exposure and noise were the most common headache-worsening factors (Table 3).

Table 4 shows 80.4% selected rest as the primary treatment. Only 15% engaged in regular exercise; 46% exercised less than 30 min per day. 4.9% had moderate sleep quality (PSQI 5–10), and 95.1% had poor sleep quality (PSQI > 10).

Chi-square analyses identified significant associations between CGH severity and: perceived stress (*p* = 0.002), prolonged device use (*p* < 0.001), headache radiating from neck to head/eyes (*p* = 0.001), exercise duration (*p* = 0.056), exercise worsening headache (*p* = 0.006), medication use (*p* < 0.001), headache tolerance without medication (*p* < 0.001), and sleep quality (*p* = 0.044) (Table 5).

## 4. Discussion

CGH is prevalent among students worldwide, affecting both physical and psychological well-being [6,26]. This study investigated the relationship between prolonged use of electronic devices, levels of physical activity, medication use, sleep quality, and self-reported cervicogenic-type head and neck pain among college students in Saudi Arabia. The one-month self-reported prevalence of head and neck pain was 65%. Given the cross-sectional design, no causal inferences can be drawn. The higher prevalence of self-reported head and neck pain observed in third to fifth year students may reflect the cumulative exposure to prolonged device use and elevated academic stress during this period, though the cross-sectional design precludes any causal interpretation. In addition, it should be noted that students in the third to fifth years of study are inherently subject to greater academic pressure and cognitive demands, which may independently elevate stress, reduce sleep quality, and increase sedentary device use, all of which are associated with headache regardless of cervicogenic etiology. Without a headache-free comparison group drawn from a similar academic population, we cannot determine whether the observed associations are specific to CGH or reflect the general profile of senior university students.

The demographic composition of our sample warrants careful consideration when interpreting these findings. Female students constituted 84.3% of respondents, which is markedly disproportionate relative to the gender distribution of the Saudi university student population as a whole (approximately 50% female according to national higher education statistics). This imbalance likely reflects the differential response propensity of female students, who may have been more willing to engage with a health-focused online questionnaire and may have amplified the observed prevalence of head and neck pain, as headache disorders, including migraine, are known to be more prevalent in women, partly due to hormonal mechanisms [27]. Additionally, gender-specific psychological stressors might account for the observed differences between the genders [26].

Our findings align with existing research that indicates a correlation between excessive electronic device usage and CGH disorders [28,29]. Students who used electronic devices for 4 or more hours daily were more frequently represented in the moderate-to-severe headache group than those reporting less than one hour of daily use. While this is consistent with prior literature linking prolonged device exposure to cervical strain and headache [30], the observed pattern cannot be interpreted causally given the cross-sectional design. Additionally, we found a strong association between physical activity and headaches, consistent with prior research indicating that exercise may help prevent headaches [31]. Potential mechanisms include stress reduction, endorphin release, and improved sleep quality [32,33]. This study is limited by its cross-sectional design and reliance on self-reported data. Additionally, we did not examine other factors, such as mental health, coffee intake, or diet. The strengths of this research include a substantial response rate and a thorough assessment of multiple facets associated with CGHs. These findings suggest that excessive electronic device use and physical inactivity are associated with greater self-reported head and neck pain severity among college students. Whether targeted interventions addressing these behaviors would reduce the occurrence of CGH requires confirmation through future longitudinal or experimental studies.

The mechanisms by which prolonged electronic device use may contribute to cervicogenic-type headache involve an interconnected cascade of biomechanical and neurophysiological processes. At the structural level, sustained forward head posture characteristic of smartphone and tablet use increases the effective gravitational load on the cervical spine from approximately 5 kg in neutral alignment to an estimated 27 kg at 45 degrees of neck flexion [34]. This compressive load accelerates fatigue in the cervical extensor musculature and promotes adaptive shortening of the suboccipital muscles, which contain a high density of muscle spindles that project directly to the trigeminocervical nucleus caudalis (TCC). At the neurophysiological level, sustained nociceptive input from sensitized cervical structures, including the C1–C3 facet joints, upper cervical myofascia, and dural afferents, converges on second-order neurons in the TCC. This convergence underpins the referral of cervical pain to the frontotemporal and periorbital regions and facilitates peripheral sensitization of cervical afferents. With repeated or chronic activation, central sensitization may develop, characterized by lowered pain thresholds, expanded receptive fields, and heightened sensitivity to normally innocuous stimuli such as light and noise, findings reflected in our results, in which light exposure and noise were the most frequently reported headache-worsening factors. Physical inactivity, documented in our sample (46% exercising less than 30 min daily), may further perpetuate these mechanisms by reducing endorphin-mediated descending pain inhibition and impairing sleep architecture itself, a modulator of central pain sensitization. This mechanistic chain, from postural load through peripheral and central sensitization to headache generation, provides a coherent physiological rationale for the associations observed in the present study and aligns with the shared sensitization model recently proposed across headache subtypes [3,4].

Over the past decade, smartphones have become integral to our daily lives, resulting in a significant rise not only in ownership but also in prolonged sedentary postures, which may lead to musculoskeletal issues [35]. Extended static positioning while using a smartphone can lead to spinal misalignment, pain, and discomfort by increasing cervical flexion, which may also exacerbate thoracolumbar pain by altering spinal curvatures and increasing activation of both contractile and non-contractile tissues [34]. Kim and Kim (2015) indicated that the cervical area is the most susceptible to adverse effects associated with smartphone usage [36]. Inal and Serel Arslan (2021) indicated that smartphone addiction may lead to detrimental postural patterns, subsequently contributing to shoulder and upper extremity pain among university students [37]. Lee (2016) reported diminished neck muscle endurance and elevated Neck Disability Index scores among university students associated with extended smartphone use [38]. It is important to acknowledge that much of the electronic device use reported by college students serves academic purposes, such as reviewing lecture notes, reading e-books, and attending online classes. The biomechanical posture adopted during academic device use (e.g., cervical flexion over a tablet or phone screen) may not differ substantially from that assumed when reading a physical textbook. Future studies should distinguish between academic and recreational device use and assess whether the nature of the task, the duration of static posture, or the type of device modifies the association with cervicogenic-type headache.

We demonstrated high levels of impaired sleep quality, with 95.1% exhibiting poor sleep quality (PSQI > 10). Sleep disturbances are more prevalent among youth due to the digitization of lifestyles [39]. Furthermore, the complete absence of participants with normal sleep quality (PSQI < 5) is an unexpected finding. While poor sleep is highly prevalent among university students and has been consistently associated with excessive screen time [40,41], a 0% rate of normal sleep is unlikely to reflect the true population distribution and may reflect a self-selection effect, whereby students experiencing the most pronounced symptoms were most motivated to participate.

Given the observational design of this study, which was limited to identifying associations rather than establishing causal pathways, programs targeting electronic device overuse and physical inactivity should be considered exploratory rather than evidence-based interventions. Confirmatory longitudinal research is necessary before firm recommendations can be made. The connections between possible triggers, such as stress, sleep problems, food habits, and headache outcomes over time, should be further investigated by longitudinal studies. Examining how variables such as age, gender, comorbidities, and other factors can influence the development of individualized treatment plans is also crucial. Intervention trials are necessary to examine the long-term effects of lifestyle changes on headache patterns, such as exercise, stress reduction, sleep hygiene, and diet. This research may clarify the best “doses” and combinations of non-pharmacological headache management and prevention techniques.

## 5. Limitations

The cross-sectional design limits our ability to determine causal relationships or temporal associations. The absence of a headache-free control group limits our ability to determine whether the observed associations are specific to cervicogenic headaches or reflect general characteristics of college students. The inherently elevated stress levels associated with university study, particularly in the third to fifth years, may independently account for the patterns observed, irrespective of headache status. The primary classification of CGH was based on self-reported co-occurrence of head and neck pain rather than ICHD-3 clinical criteria. As a result, the true proportion of participants who meet formal CGH diagnostic criteria is unknown, and the sample may include individuals with migraine, tension-type headache, or mixed presentations. This limits the diagnostic specificity of our findings and represents the most significant methodological constraint of the current study. We did not assess other potentially significant factors such as caffeine intake, dietary triggers, or smoking. The study has several notable sampling limitations. First, 84.3% of participants were female, which is disproportionate relative to the general Saudi university student population and likely inflated the estimated prevalence of head and neck pain. Second, not a single participant reported normal sleep quality (PSQI score < 5), a finding that almost certainly reflects a self-selection effect rather than a true population characteristic. Participants were recruited via convenience sampling from a limited number of universities, introducing selection bias and limiting the representativeness of the estimated prevalence. The observed rate of 65.2% should therefore be considered a sample-specific figure rather than a population-level prevalence estimate for Saudi university students. Future studies employing stratified random sampling across a nationally representative range of institutions are needed to produce reliable prevalence estimates. The absence of formal psychometric validation for the non-PSQI questionnaire components is also acknowledged as a limitation.

## 6. Conclusions

This study identifies significant associations between self-reported cervicogenic-type head and neck pain and excessive electronic device use, physical inactivity, medication reliance, and poor sleep quality among Saudi college students. These associations highlight potential targets for future interventional research. However, given the cross-sectional design, no causal inferences can be drawn. Longitudinal studies and randomized controlled trials are needed to determine whether modifying these behaviors reduces the burden of cervicogenic-type headache in this population.

### Recommendations

Based on the associations identified in this study, the following research and practice directions are proposed:

1. Future longitudinal studies should investigate whether reducing daily electronic device use is prospectively associated with lower incidence or severity of cervicogenic-type headache among college students.
2. Intervention trials examining the effect of structured physical activity programs on self-reported head and neck pain in device-heavy student populations are warranted.
3. Universities may consider developing ergonomic awareness programs; however, the effectiveness of these programs in reducing CGH should be evaluated through controlled study designs before widespread implementation.
4. Future research should incorporate objective measures of device use, clinically verified CGH diagnoses (ICHD-3 criteria), and actigraphy-based sleep assessment to strengthen the evidence base.
5. Dietary habits, psychosocial determinants, and quality of life should be assessed in future multifactorial studies to develop a comprehensive understanding of the contributing factors to CGH.

## Figures and Tables

**Table 1:** Demographic characteristics of the respondents (N = 313).

Characteristic	n	%
**Gender**		
Male	49	15.7
Female	264	84.3
**Age (Years)**		
18–21	176	56.2
22–25	122	39.0
26–30	10	3.2
31 and above	5	1.6
**Marital status**		
Single	295	94.2
Married	18	5.8
**Academic Year**		
First	53	16.9
Second	45	14.4
Third	50	16.0
Fourth	89	28.4
Fifth year or above	48	15.3
Postgraduate	28	8.9
**BMI Categories**		
cervicogenic headahce	41	13.1
Normal weight	208	66.5
Over weight	52	16.6
Obese	12	3.8
**Region in Saudi Arabia**		
Southern	39	12.5
Central	37	11.8
Eastern	14	4.5
Western	60	19.2
Northern	163	52.1
**Painful body regions**		
Head and neck	204	65.2
Upper back	27	8.6
Lower back	61	19.5
Shoulder	12	3.8
Elbow	0	0
Wrist & Fingers	3	1.0
Hip	1	0.3
Knee	3	1.0
Ankle & Foot	2	0.6
**Duration of the problem**		
Less than a week	24	7.7
1 week to 1 month	82	26.2
2 months to 6 months	100	31.9
7 months to 1 year	29	9.3
more than 1 year	78	24.9
**Perceived stress level**		
Mild	136	43.5
Moderate	150	47.9
Severe	27	8.6

**Table 2:** Electronic device usage among students with head and neck pain (n = 204).

Electronic Devices Used (Daily)	n (n = 204)	%
Smartphone	132	64.7
Tablet device (Tablet or iPad)	48	23.5
Computer	23	11.3
Smart TV	1	0.5
**Primary devices use duration (hours/day)**		
Less than 1 h	2	1.0
1–2 h	9	4.4
3–4 h	34	16.7
More than 4 h	159	77.9
**All devices use duration (hours/day)**		
Less than 1 h	6	2.9
1–2 h	15	7.4
3–4 h	24	11.8
More than 4 h	159	77.9
**Rate of use per session**		
Continuously	117	57.4
Without stopping	11	5.4
Interrupted	75	36.8
I don’t use devices continuously	1	0.5

**Table 3:** Headache patterns and characteristics after electronic device use (n = 204).

Headache Severity (Past Week/Months)	n (n = 204)	%
1–3 (mild pain)	19	9.3
4–6 (moderate pain)	105	51.5
7–10 (severe pain)	80	39.2
**Headache duration**		
Less than 30 min	73	35.8
31 min to 1 h	55	27.0
1–2 h	42	20.6
More than 2 h	34	16.7
**Headache medications (last 30 days)**		
1–5 days	78	38.2
6–10 days	11	5.4
More than 10 days	12	5.9
I do not take any pills	103	50.5
**Duration without medicaion**		
Less than 30 min	113	55.4
31 min to 1 h	0	20.6
1–2 h	42	24.0
More than 2 h	49	55.4
**Headache location**		
Left/Right side	57	27.9
Both sides	42	20.6
Forehead	64	31.4
Back of the head	41	20.1
**Type of headache**		
Throbbing or pulsating	58	28.4
Pressing or squeezing	59	28.9
Sharp or stabbing	13	6.4
Every attack with different pattern	74	36.3
**Triggers/worsening factors**		
Exercises	0	0.0
Light exposure	68	33.3
Noise	98	48.0
Other	38	18.6

**Table 4:** Physical activity and sleep quality among students with head and neck pain (n = 204).

Primary Treatment for Headache/Neck Pain	Number (n = 204)	Percentage (%)
Rest	164	80.4
Consulting Doctor	9	4.4
Physical Therapy	18	8.8
Other	13	6.4
**Physical activity frequency**		
Always	34	16.7
Usually	58	28.4
Sometimes	77	37.7
Never	35	17.2
**Regular exercise**		
Always	30	14.7
Usually	42	20.6
Sometimes	74	36.3
Never	58	28.4
**Exercise days per week?**		
One day	86	42.2
2–3 days	71	34.8
4–6 days	35	17.2
Everyday	12	5.9
**Exercise duration per day**		
Less than 30 min	94	46.1
31–60 min	69	33.8
61–120 min	39	19.1
More than 120 min	2	1.0
**Does exercise worsen headache?**		
Always	17	8.3
Usually	33	16.2
Sometimes	68	33.3
Never	86	42.2
**Known physical therapy measures**		
TENS	13	6.4
ULTRA	6	2.9
Stretching	81	39.7
Exercise	76	37.3
Strengthening	7	3.4
Patient Education	17	8.3
Other	4	2.0
**Sleep Quality (PSQI score)**		
Normal Sleep Quality (Less than 5)	0	0.0
Moderate Sleep Quality (5 to 10)	10	4.9
Poor Sleep Quality (more than 10)	194	95.1

**Table 5:** Chi-square associations between headache severity and key variables (n = 204).

Variable	Mild Pain (0–3)		Moderate Pain (4–6)		Severe Pain (7–10)		Chi-Square	*p*
**Perceived stress level**								
Mild	11	13.3	51	61.4	21	25.3	16.684 ^a^	0.002 *
Moderate	4	4.1	47	48.0	47	48.0		
Severe	4	17.4	7	30.4	12	52.2		
**Neck pain/headache after prolonged device use**								
Always	3	5.6	28	51.9	23	42.6	26.723 ^a^	0.000 *
Usually	4	4.9	43	52.4	35	42.7		
Sometimes	7	11.9	32	54.2	20	33.9		
Never	5	55.6	2	22.2	2	22.2		
**Headache start from neck and spread to the head/eyes**								
Always	0	0.0	27	37.8	28	62.2	23.370 ^a^	0.001 *
Usually	2	3.8	29	55.8	21	40.4		
Sometimes	11	15.1	38	52.1	24	32.9		
Never	6	17.6	21	61.8	7	20.6		
**Exercise days per week?**								
One day	6	7.0	43	50.0	37	43.0	12.290 ^a^	0.056 *
2–3 days	6	8.5	41	57.7	24	33.8		
4–6 days	3	8.6	15	42.9	17	48.6		
Everyday	4	33.3	6	50.0	2	16.7		
**Does exercise worsen headache?**								
Always	1	5.9	10	58.8	6	35.3	17.988 ^a^	0.006 *
Usually	1	3.0	16	48.5	16	48.5		
Sometimes	3	4.4	29	42.6	36	52.9		
Never	14	16.3	50	58.1	22	25.6		
**Medication use (last 30 days)**								
1–5 days	1	1.3	40	51.3	37	47.4	31.820 ^a^	0.000 *
6–10 days	0	0.0	1	9.1	10	90.9		
More than 10 days	0	0.0	7	58.3	5	41.7		
I do not take any pills	18	17.5	57	55.3	28	27.2		
**Headache duration without medication)**								
Less than 30 min	16	14.2	62	54.9	35	31.0	20.976 ^a^	0.000 *
31 min to 1 h	0	0.0	0	0.0	0	0.0		
1–2 h	1	2.4	27	64.3	14	33.3		
More than 2 h	2	4.1	16	32.7	31	63.3		
**Sleep Quality**								
Moderate Sleep Quality (5–10)	3	30.0	5	50.0	2	20.0	5.826 ^a^	0.044 *
Poor (>10)	16	8.2	100	51.5	78	40.2		

* *p* < 0.05 (statistically significant). ^a^ Chi-square values shown for first category only within each grouping variable.

## Data Availability

All data associated with the research study are presented in the manuscript.
